# Supplementary figures and images for: Identification of Zinc-Dependent Mechanisms Used by Group B Streptococcus To Overcome Calprotectin-Mediated Stress
Source: mBio. 2020 Nov 10;11(6):e02302-20. doi: 10.1128/mBio.02302-20 (PMC7667036; doi:10.1128/mBio.02302-20)

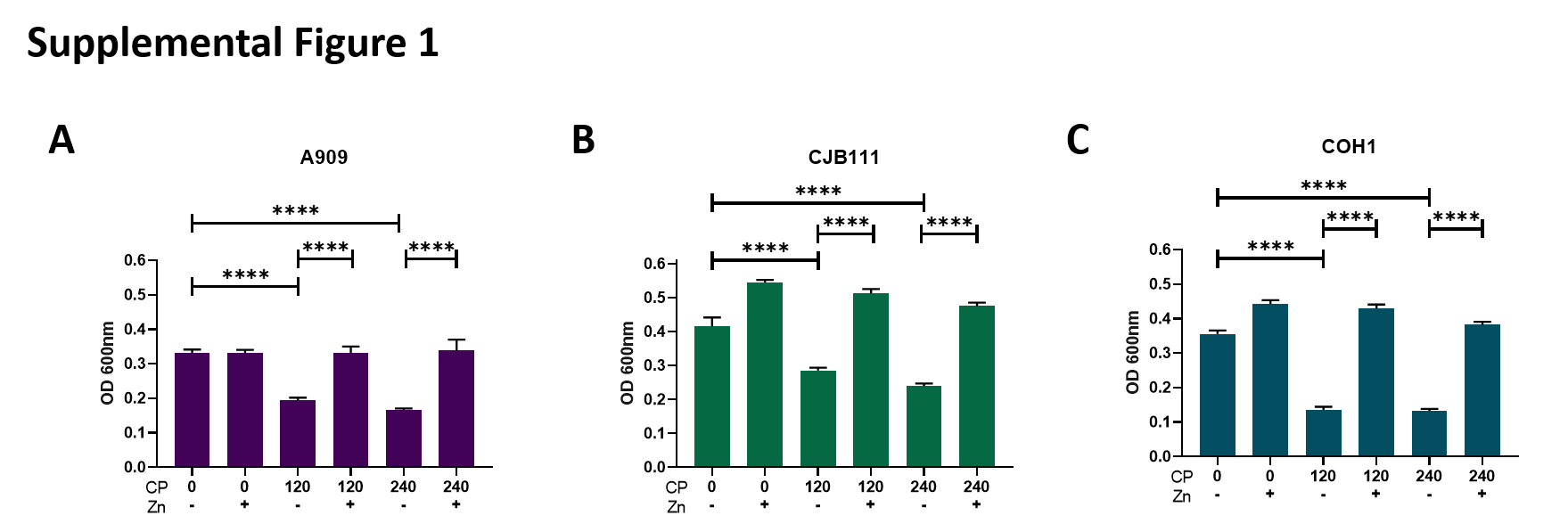

Supplement: FIG S1 [file mBio.02302-20-sf001.tif]

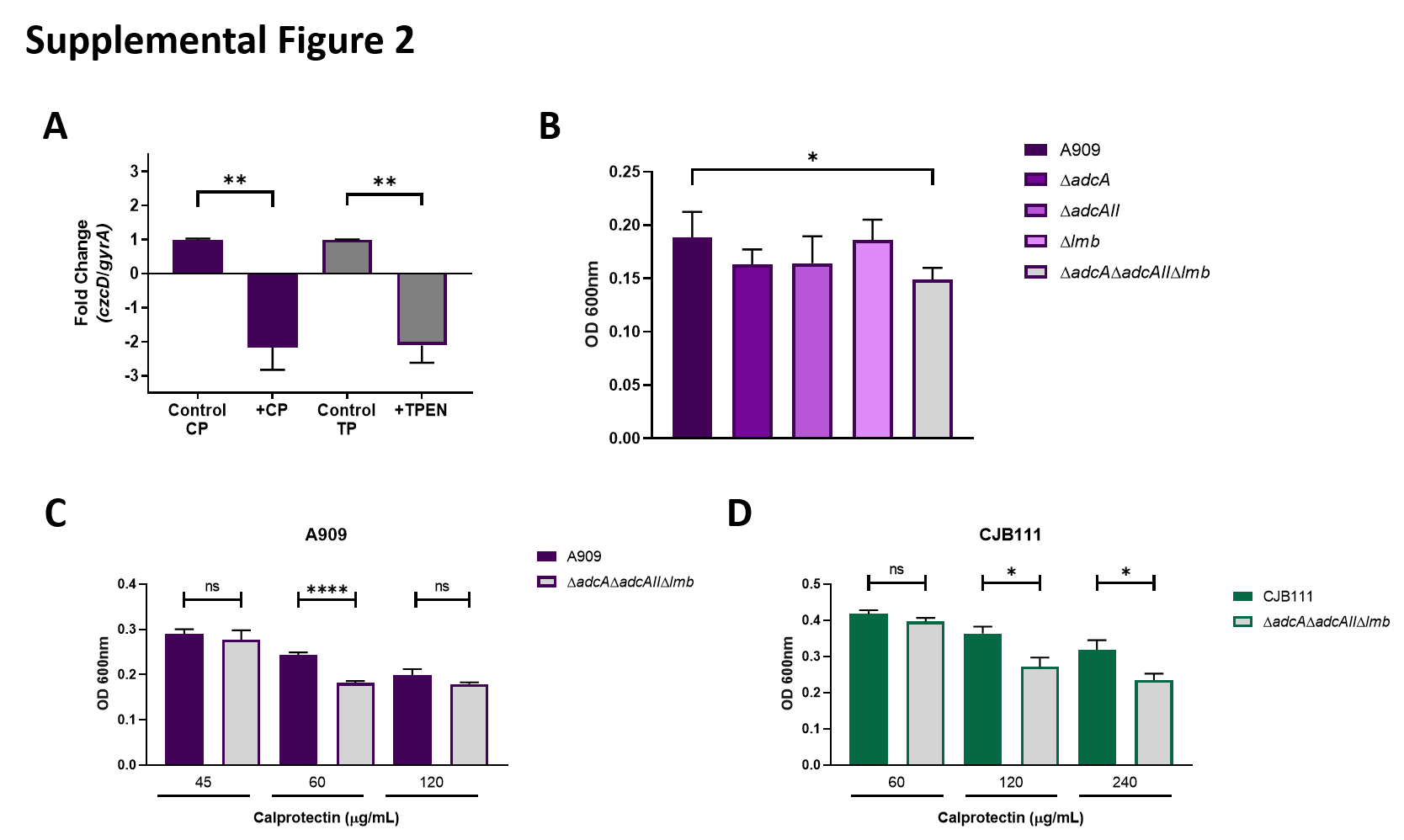

Supplement: FIG S2 [file mBio.02302-20-sf002.tif]

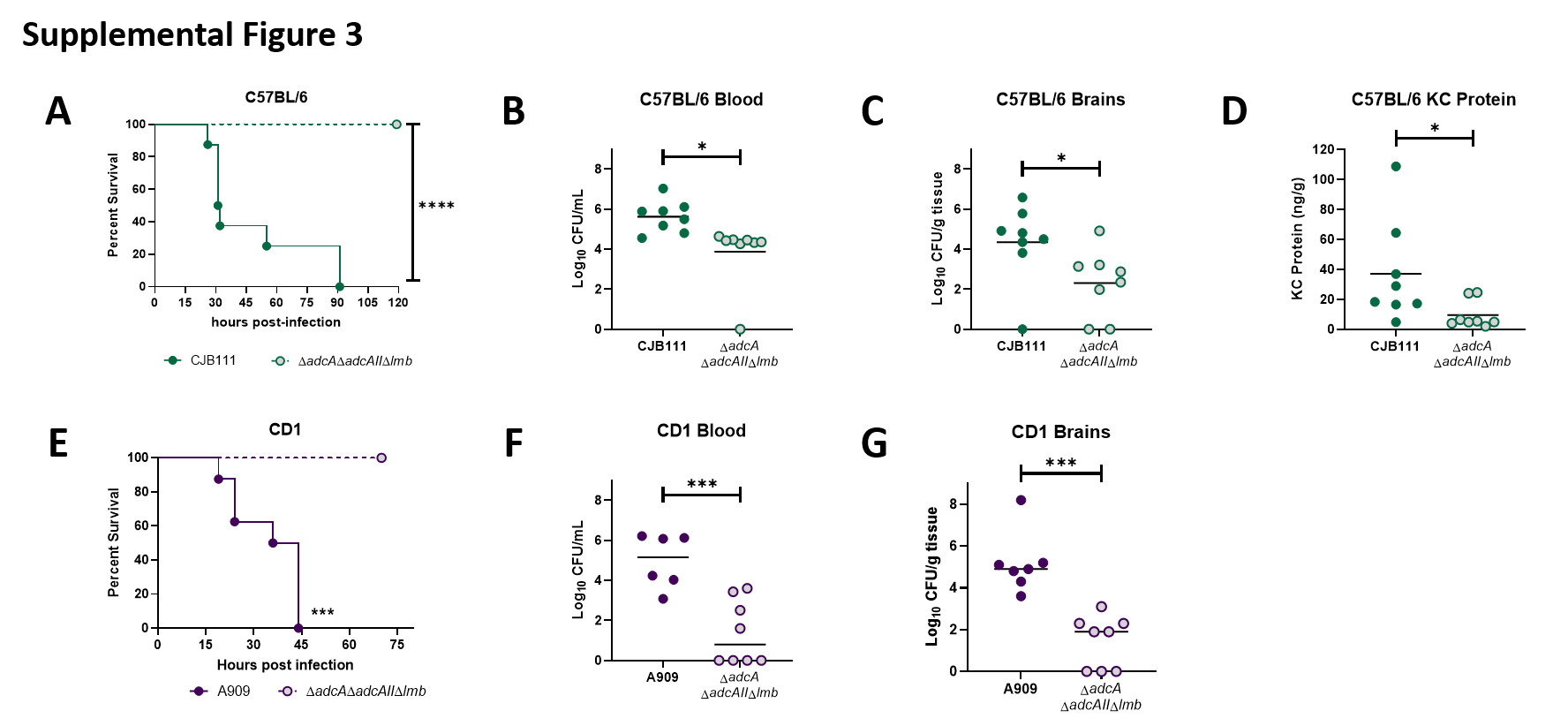

Supplement: FIG S3 [file mBio.02302-20-sf003.tif]
